# Random repeated cross sectional study on breeding site characterization of *Anopheles sinensis *larvae in distinct villages of Yongcheng City, People's Republic of China

**DOI:** 10.1186/1756-3305-5-58

**Published:** 2012-03-23

**Authors:** Xiao-Bo Liu, Qi-Yong Liu, Yu-Hong Guo, Jing-Yi Jiang, Dong-Sheng Ren, Guang-Chao Zhou, Can-Jun Zheng, Jing-Li Liu, Yun Chen, Hong-Sheng Li, Hua-Zhong Li, Qun Li

**Affiliations:** 1 State Key Laboratory for Infectious Disease Prevention and Control, China CDC Key Laboratory of Surveillance and Early-Warning on Infectious Disease, National Institute for Communicable Disease Control and Prevention, Chinese Center for Disease Control and Prevention, Beijing 102206, China; 2 Yongcheng Center for Disease Control and Prevention, Yongcheng 476600, China; 3 Office for Disease Control and Emergency Response, Chinese Center for Disease Control and Prevention, Beijing 102206, China

**Keywords:** Breeding site, Characterization, Mosquito, *Anopheles *vectors, Ecology, Malaria elimination

## Abstract

**Background:**

Characterizing the breeding site of *Anopheles sinensis *is of major importance for the transition from malaria control to elimination in China. However, little information is available especially regarding the characteristics and influencing factors of breeding sites of *An. sinensis *in Yongcheng City, a representative region of unstable malaria transmission in the Huang-Huai River region of central China. The aims of this study were to determine the breeding site characteristics of *An. sinensis *and related environmental and physicochemical parameters, to find out which breeding site characteristics could best explain the presence of *An. sinensis *larvae, and to determine whether the breeding habit of *An. sinensis *has changed or not.

**Methods:**

Random repeated cross sectional study was undertaken in six villages of the Yongcheng city characterized by different levels of the historical incidence of *P. vivax *malaria. The potential breeding sites of *An. sinensis *larvae in each village were examined twice per month both in the household courtyards and the village surroundings. The larval sampling was done by the standard dipping method. Some important breeding site characterizations were recorded and characterized. The anopheline mosquito larvae and emerged adults were identified to the species level morphologically and to sub-species by the ribosomal DNA PCR technique. Chi-square analysis and logistic regression analysis were applied to determine the importance of factors for explaining the presence or absence of *An. sinensis *larvae.

**Results:**

According to the ribosomal DNA PCR assay, all sampled anopheline mosquito larvae and emerged adults belonged to *An. sinensis*. Only 3 containers that were sampled from the household courtyards were found to contain *An. sinensis *larvae. There were no differences in the species composition of mosquito larvae among containers that contained water in the household courtyards (P > 0.05). *An. sinensis *larvae were shown to be present in a total of 60 breeding sites in the village surroundings, this included 8 (13.3%) river fringes, 26 (43.3%) ponds, 23 (38.3%) puddles, and 3 (5.0%) irrigation/drainage ditches. Logistic regression analysis revealed that the breeding site type, water depth, chemical oxygen demand (COD), ammonia nitrogen, and sulphate were found to be the key factors determining the presence of *An. sinensis *larvae. Approximately 94.9% of *An. sinensis *larvae inhabited relatively large and medium-sized water bodies, with depths between 0.5 m and 1.0 m (73.3%), COD lower than 2 mg/L (75%), ammonia nitrogen lower than 0.4 mg/L (86.7%), and sulphate lower than 150 mg/L (58.3%), respectively.

**Conclusion:**

These results indicate that the majority of *An. sinensis *larval breeding sites were relatively large and medium-sized water bodies with depths between 0.5 m and 1.0 m, and containing low levels of COD, ammonia nitrogen, and sulphate, respectively. For effective *An. sinensis *larval control, the type of breeding site, water depth, COD, ammonia nitrogen, and sulphate should be given higher priority over other factors in areas where it is the primary vector.

## Background

Malaria is a major health and economic threat to about 40% of the world's population [1-3]. *Plasmodium falciparum *malaria is responsible for the majority of the disease burden [4-6], while *Plasmodium vivax *malaria is the geographically most widely distributed [7,8]. The current estimate of human lives at risk from *P. vivax *malaria is 2.6 billion [9,10], and South and East Asia account for 52% of the total *P. vivax *malaria burden [11]. Re-emergence of malaria often becomes a serious public health threat in many countries [12]. In China, malaria has been prevalent throughout the country for many years [13], and still represents a major public health problem [14,15]. Cases of malaria have dramatically increased in the areas along the Huang-Huai River region of central China after 2001, especially in Henan Province and Anhui Province [14,16-18]. In response to the global initiative to eradicate malaria [19-24], a national plan for malaria elimination was proposed by the Chinese Ministry of Health in 2009, and a nationwide campaign was launched by the Chinese Government in 2010, to eliminate malaria in most endemic regions by 2015 and to achieve ultimate national elimination by 2020. In China, *Anopheles sinensis*, *Anopheles lesteri*, *Anopheles minimus *and *Anopheles dirus *are considered to be the principal vectors of malaria [25-27]. However, malaria outbreaks and re-emergences were only in the areas where *An. sinensis *is the principal vector in recent years [14,28,29].

Each anopheline mosquito species has its preferred breeding site for oviposition, depending on weather conditions, physical geography and human activity. Breeding sites could be natural or man-made, shaded or sunny, permanent or temporary, of various sizes and located in running or stagnant water bodies, among others [30,31]. Previous studies have demonstrated that *An. sinensis *larvae were commonly found in shallow and fresh water breeding sites with emergent vegetation, and exposure to sunlight [32]. The main breeding sites include rice fields, open grass ponds, irrigation canals, ditches, ground pools, swamps, marshes, streams, shores of lakes, stream margins, and seepages, among others. Environmental factors in the breeding sites are closely related to the breeding habit of the mosquito larvae. The changes of physical factors (temperature, light, water, and wave), chemical factors (chloride content and organic pollution), and biological factors (aquatic vegetation, food, and natural enemy), may cause the changes in breeding habit of mosquito larvae. However, from the existing literature little is known about the change of breeding habit of *An. sinensis *in the regions of unstable malaria transmission in China. In recent years, some factors, such as the local ecological alterations due to the increasing water pollution [33], the adjustment of regional agricultural structures [34], the increased use of chemical pesticides [35,36], and the changing global climate [37-44], can significantly affect the population dynamics and the breeding habits of *An. sinensis*, indirectly affect the transmission of *P. vivax *malaria [14,45,46], and pose a significant challenge to the implementation and achievement of malaria elimination in China.

Informed larval interventions that target more prolific breeding sites have enormous potential in combating *P. vivax *malaria, especially at a regional scale. Therefore, characterizing *An. sinensis *breeding sites and related environmental and physicochemical factors could be very useful in understanding the variations observed in malaria transmission intensity, so that more effective vector control strategies could be planned. However, little information is available regarding the precise breeding sites and the changes of breeding habit of *An. sinensis *in Yongcheng City, the most highly endemic area in the Huang-Huai River region of central China in recent years. Therefore, the main objectives of the present study were to determine the breeding site characteristics and related environmental and physicochemical factors of *An. sinensis*, in order to find out which breeding site characteristics could best explain the presence of *An. sinensis *larvae, and to determine whether the breeding habit of *An. sinensis *has changed or not. The results can provide baseline data essential for planning and implementing of *An. sinensis *larval abatement programs in China.

## Methods

### Study area

**Figure 1:**
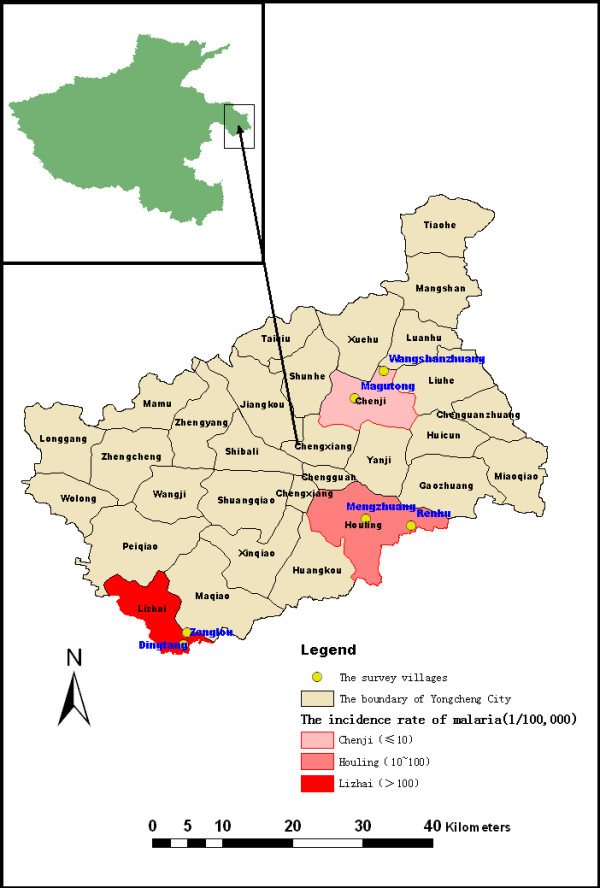
**Map showing the survey villages in Yongcheng city of Henan Province, People's Republic of China**. Yellow dot represents the study village; Dingtang village and Zenglou village in Lizhai township belongs to high levels of historical incidence of *P. vivax *malaria; Renhu village and Mengzhuang village in Houling township belongs to intermediate level of historical incidence of *P. vivax *malaria; Wangshanzhuang village and Magutong village in Chenji township belongs to low levels of historical incidence of *P. vivax *malaria.

The study was undertaken in six villages of the Yongcheng city characterized by different levels of the historical incidence of *P. vivax *malaria. These included two high risk villages, namely Dingtang village and Zenglou village (Lizhai township, annual average incidence rate > 100/100,000), two intermediate risk villages, namely Renhu village and Mengzhuang village (Houling township, annual average incidence rate 10 ~ 100/100,000), and two low risk villages, namely Wangshanzhuang village and Magutong village (Chenji township, annual average incidence rate < 10/100,000) [47], (Figure 1). Among them, *P. vivax *malaria was endemic and transmitted by *An. sinensis *[34].

These villages are located in an area with the latitudes between 33°42' and 34°18', and longitudes between 115°58' and 116°39' [34]. Most of the regions are flat at about 33 meters high altitude. The climate is warm temperate from May to October, and the annual average temperature is 14.3°C. The range of annual rainfall is between 556.2 mm and 1,648.9 mm, and the dense rainfall is concentrated in June to September. The Tuohe River is the main environmental feature in the area. These rivers break into small ponds and puddles during the hot and dry season, and ponds and puddles, which are formed by the river become adequate breeding sites of *An. sinensis*. The inhabitants of these villages live in brick houses. In these villages, the primary farm crops include wheat, soybean, corn and a small amount of cotton and potato. During summer, most of local residents tend to sleep outdoors [26].

Besides the difference in the level of the historical incidence of *P. vivax *malaria, other characteristic differences between the six studied villages are as follows: First, Dingtang village and Zenglou village are adjacent to Guoyang County; Renhu village and Mengzhuang village are neighbouring to Suixi County. Guoyang County and Suixi County are unstable regions of *P. vivax *malaria in Anhui Province. In contrast, Wangshanzhuang village and Magutong village are not adjacent to Anhui Province. Second, the water-body distributions and appropriate breeding sites of *An. sinensis *larvae in Dingtang village, Zenglou village, Renhu village, and Mengzhuang village are more than those in Wangshanzhuang village and Magutong village. Third, the population of animal hosts in Renhu village, Mengzhuang village, Zenglou village, and Dingtang village was larger than that of Wangshanzhuang village and Magutong village [34].

### Mosquito larvae sampling and identification

Random repeated cross-sectional study was carried out from July 1^st ^to November 30^th^, 2010. The breeding sites in each village were examined twice per month for the presence of aquatic stages of anopheline and culicine mosquito species. Each village was systematically searched for the potential breeding sites both in the household courtyard and in the village surroundings. The larval sampling was done by the standard dipping method as described by Service [48]. In the household courtyard of each village, 10 household courtyards were randomly sampled. The primary container types in the household courtyard are of great epidemiologic significance, if a high density of *An. sinensis *larvae inhabited these types of containers. Therefore, all the containers in the sampled household courtyard were inspected for the presence of mosquito larvae and pupae during the study period. In the village surroundings, all the potential breeding sites within 1 km were located and inspected. When mosquito larvae were present, 10 dips were taken with a dipper in each breeding site. When a breeding site was too small to make 10 dips, water was dipped as many times as possible [49]. In extensive water bodies, dipping was carried out at 100 m apart. The pupae were also collected and transferred into a bottle beaker containing 250 ml water obtained from the breeding site. The pupae were then taken into the laboratory in Yongcheng CDC, and allowed to emerge.

In the field of each village, anopheline mosquito larvae were separated from culicine larvae and *Aedes *larvae. The anopheline mosquito larvae were classified as early instar stages (I and II) and late instar stages (III and IV) according to the keys of Gillies and Coetzee [50]. A portion of the late instars of anopheline mosquito larvae were immediately preserved in 90% absolute ethanol and then taken to the laboratory of the Chinese Center for Disease Control and Prevention (China CDC) for species identification under a compound microscope using commonly accepted guidelines [51,52]. The sampled anopheline mosquito larvae were preserved individually in Eppendorf tubes containing absolute ethanol, pending further identification by polymerase chain reaction (PCR). A Qia Amp DNA Mini Kit (Qiagen Inc., CA) was used and DNA was extracted from these late instar larvae and thorax of emerged *An. sinensis *according to the manufacturer's instructions. The PCR conditions used in the present study were the same as a study of Ma Yajun, *et al. *in China [53]. The late stage instars of anopheline mosquito larvae and emerged adults were identified to the species level morphologically, using taxonomic keys and to sub-species by the rDNA PCR technique [53-55], because individual species within the Hyrcanus Complex could not be identified by morphology alone.

### Larval breeding site characterization

Some important breeding site characterizations, namely container type, water depth, substrate type, canopy, and surface debris were recorded for all the containers in the household courtyard. In the village surroundings, the larval breeding sites were characterized either visually or using hand-held equipment [56]. The primary container types were classified as follows: plastic bucket (a plastic container and used for containing water), cement crock (a cement container with a larger opening and used for containing water and grain), stone groove (a stone rectangular container used for feeding pigs, chickens, goats, sheep, among others), washbasin (plastic or metal container used for washing face), tile jar (a small ceramics jar used as a container for containing water and grain), bowl, tray, and gallipot (a small earthenware pot used by pharmacists as a container for ointments). The identified bodies of water were classified according to their nature: river fringes (breeding sites formed along river banks when the water level drops), ponds (water area larger than 50 m^2^), puddles (water area less than 50 m^2^), irrigation/drainage ditches, and ground pools [56]. The perimeter of each breeding site was categorized by the estimation as shorter than 10 m, 10 ~ 100 m, and longer than 100 m [57]. Substrate types were classified into muddy, sandy (gravel with soil), and cinder. Distance to the nearest house was measured by GPS. Water depth was classified into shorter than 0.5 m, 0.5 ~ 1.0 m, and longer than 1.0 m. Canopy was measured visually by estimating the area of larval breeding site covered by shade, terrestrial vegetation, and other objects [58]. Surface debris was also estimated visually by researchers. In each village, the stability of mosquito larval breeding sites was classified as either temporary or permanent. Temporary breeding sites held water for a short period of time (until approximately two weeks after the rainy season ended) and stemmed mainly from rain showers. When rain ceased these breeding sites dried out. The permanent breeding sites, on the other hand, held water for a longer period of time (approximately two to three months after the rain ended or fed by natural underground sources) and hence were more stable [12]. The permanent breeding sites remained in the same location throughout the sampling period while temporary breeding sites changed depending on the availability of water. In the present study, permanent breeding sites found during the study period included river fringes, ponds and puddles. Temporary breeding sites included roadside ditches, rain pools, shallow drainages, and ground pools.

In each locality, water samples were collected for physico-chemical analysis. Water samples were transferred to the laboratory in tightly closed glass bottles and kept at 2 -8°C in a refrigerator. They were analyzed within a maximum of 1 day post-collection [59]. Physico-chemical analysis of water from the *Anopheles *mosquito breeding site was done in the laboratory of the Yongcheng CDC. Physical factors include turbidity and chromaticity. Turbidity was measured by transmissometer and was classified into two levels as less than 0.3 and larger than 0.3. Chemical factors include pH, chemical oxygen demand (COD), ammonia nitrogen, sulphate, and chloride. The pH value was measured by an electronic device (HANNA^©^HI 98128 Combo PH&EC, Hanna Instruments. US DESIGN PATENT D462, 024). The contents of COD, ammonia nitrogen, sulphate, and chloride in the water obtained from *An. sinensis *breeding sites were measured in the laboratory of Yongcheng CDC, using standard methods [60]. Biological factors studied in the present study include predator, algae cover, emergent and floating plants, among others. Predators include larvivorous fishes, dragonfly larvae, water scorpions, water bugs, among others. The algae cover was estimated as the percentage of the total breeding site [56]. Emergent plants included both aquatic and immersed terrestrial vegetation. All visual classifications were performed by one person throughout the sampling period to avoid discrepancies.

### Ethical considerations

Ethical approval for this study was obtained from the Ethical Committee of China CDC and permission was also obtained from the Municipal Government, the Municipal Health Bureau in Yongcheng city and Yongcheng CDC.

### Data analysis

The composition of mosquito larvae among containers that contained water in the household courtyard was calculated. A Chi-square analysis was applied to determine the importance of factors for explaining the presence or absence of *An. sinensis *larvae. Some factors which are of statistical significance using Chi-square analysis, were selected and further analyzed by Binary logistic regression analysis to calculate the odds ratio (OR) and 95% Wald Confidence Intervals. However, this study focuses on the presence of *An. sinensis *larvae rather than their abundance in the aquatic environment, because quantification of larval abundance is prone to sampling errors, particularly in the large aquatic environment [49]. All measured parameters were included in the Binary logistic regression model. Presence of larvae was categorized as one, while the absence of larvae was categorized as zero in the logistic regression model. The water temperature, although important for the development of the mosquitoes, is only useful when the temperature range during the day is measured. However, water temperature in the present study was not discontinuously measured, thus was not included in Chi-square analysis and Binary logistic regression analysis. Statistical analysis was carried out using SPSS software (Version 11.5 for windows, SPSS Inc., Chicago, USA).

## Results

### *Anopheles *mosquito species composition

A total of 100 *Anopheles *larvae and 100 adults emerged from pupae collected in six villages, were further identified to sub-species level by the rDNA PCR assay. Based on rDNA PCR analysis, all sampled *Anopheles *larvae and adults belonged to the same species, namely *An. sinensis*. It was probably that there were no other species of the *Anopheles *mosquito of Hyrcanus Complex, except for *An. Sinensis*, that existed in Yongcheng City [61,62].

### Primary container types and breeding containers of *An. sinensis *larvae in the household courtyards

As far as the percent composition of primary container types in the household courtyards in Yongcheng city is concerned, the present study demonstrated that a plastic bucket was the most common container (41.27%), followed by cement crock (22.89%), washbasin (13.86%), and stone groove (8.13%), respectively. A small proportion of container types, such as tile jar (2.41%), bowl (1.51%), tray (1.20%), and gallipot (2.41%), also existed.

A total of 430 containers in six villages were examined, and 98 (22.8%) containers were dry at the time of visit. Among the 332 (77.2%) containers, which contained water, *An. sinensis *larvae were present in only 3 (0.90%) of these containers (Table 1). There was no difference in the composition of mosquito larvae among containers that contained water in the household courtyards (*χ*^2 ^= 46.390, df = 48, P = 0.539). As seen from Table 1, no mosquito larvae inhabited 145 (43.7%) containers which contained water, 95(28.6%)of which were productive for culicine larvae, 48 (14.5%) were productive for Aedes larvae, 7 (2.1%) were productive for *Armigeres subalbatus *larvae, and 1 (0.3%) was productive for *An. sinensis *larvae (a plastic bucket). *An. sinensis *larvae established a symbiotic relationship with culicine larvae in 2 containers (a stone groove and a cement crock) (0.6%). The findings mentioned above demonstrated that a low level of symbiotic relationship between *An. sinensis *larvae and culicine larvae was observed in these containers.

**Table 1:** Main breeding containers of anopheline and other mosquito larvae in the household courtyards in Yongcheng city, Henan Province

Container type	The number of containers that contained the following mosquito larvae(%)							Total of container
		
	Not containing mosquito larvae	*Culicine* larvae	*Anopheline *larvae	*Aedes* larvae	*Armigeres subalbatus *larvae	*Anopheline *+ *Culicine *larvae	*A*edes + *C*ulicine larvae	
**Stone groove**	14 (51.9%)	7 (25.9%)	0 (0.0%)	3 (11.1%)	0 (0.0%)	1 (3.7%)	2 (7.4%)	27 (8.13%)
**Washbasin**	27 (58.7%)	15 (32.6%)	0 (0.0%)	3 (6.5%)	0 (0.0%)	0 (0.0%)	1 (2.2%)	46 (13.86%)
**Plastic bucket**	58 (42.3%)	38 (27.7%)	1 (0.7%)	25 (18.2%)	4 (2.9%)	0 (0.0%)	11 (8.0%)	137 (41.27%)
**Cement crock**	26 (34.2%)	23 (30.3%)	0 (0.0%)	11 (14.5%)	2 (2.6%)	1 (1.3%)	13 (17.1%)	76 (22.89%)
**Tile jar**	0 (0.0%)	4 (50.0%)	0 (0.0%)	2 (25.0%)	0 (0.0%)	0 (0.0%)	2 (25.0%)	8 (2.41%)
**Bowl**	2 (40.0%)	2 (40.0%)	0 (0.0%)	1 (20.0%)	0 (0.0%)	0 (0.0%)	0 (0.0%)	5 (1.51%)
**Tray**	3 (75.0%)	0 (0.0%)	0 (0.0%)	1 (25.0%)	0 (0.0%)	0 (0.0%)	0 (0.0%)	4 (1.20%)
**Gallipot**	3 (37.5%)	1 (12.5%)	0 (0.0%)	1 (12.5%)	1 (12.5%)	0 (0.0%)	2 (25.0%)	8 (2.41%)
**^1^Others**	12 (57.1%)	5 (23.8%)	0 (0.0%)	1 (4.8%)	0 (0.0%)	0 (0.0%)	3 (14.3%)	21 (6.33%)
**Total of container**	145 (43.7%)	95 (28.6%)	1 (0.3%)	48 (14.5%)	7 (2.1%)	2 (0.6%)	34 (10.2%)	332 (100%)

^1 ^Others refer to mortar, bottle, and septic tank.

### Positive breeding sites and key influencing factors determining the presence of *An. sinensis *larvae in the village surroundings

A total of 156 potential mosquito breeding sites in the village surroundings of six villages were examined, 22.4% (35) were dry at the time of visit. Among the 121 sites which contained water, 49.6% (60) were productive for *An. sinensis *larvae. *An. sinensis *larvae were discovered in 60 breeding sites, this included 8 (13.3%) river fringes, 26 (43.3%) ponds, 23 (38.3%) puddles, and 3 (5.0%) irrigation/drainage ditches. Further biological and physicochemical factors of these breeding sites were measured, as shown in Table 2.

**Table 2:** The frequency and percent composition of the main characteristics of 121 potential mosquito breeding sites in the village surroundings

Characteristics^1^	N^2^	%^3^	Characteristics^1^	N^2^	%^3^
**The types of breeding site**			**Algae**		
river fringe	9	7.4	yes	107	88.4
pond (> 50 m^2^)	44	36.4	no	14	11.6
Puddle(< 50 m^2^)	51	42.1	**pH**		
irrigation/drainage ditch	12	9.9	< 8	23	19.0
temporary ground pool	5	4.2	8~9	60	49.6
**Perimeter**			9~10	37	30.6
< 10 m	15	12.4	> 10	1	0.8
10~100 m	79	65.3	**COD**		
> 100 m	27	22.3	< 1 mg/l	24	19.8
**Distance to the nearest house**			1~2 mg/l	37	30.6
< 10 m	50	41.3	2~3 mg/l	47	38.8
10~60 m	66	54.5	> 3 mg/l	13	10.8
> 60 m	5	4.2	**Ammonia nitrogen**		
**Substrate types**			< 0.3 mg/l	37	30.6
muddy	87	71.9	0.3~0.4 mg/l	26	21.5
sandy (gravel with soil)	30	24.8	0.4~0.5 mg/l	33	27.3
cinder	4	3.3	> 0.5 mg/l	25	20.6
**Canopy**			**Sulphate**		
yes	36	29.8	< 150 mg/l	52	43.0
no	85	70.2	150~200 mg/l	10	8.3
**Debris**			200~250 mg/l	22	18.2
yes	50	41.3	> 250 mg/l	37	30.5
no	71	58.7	**Chloride**		
**The stability of mosquito larval breeding sites**			< 15 mg/l	26	21.5
temporary	99	81.8	15~30 mg/l	57	47.1
permanent	22	18.2	30~45 mg/l	35	28.9
**Turbidity**			> 45 mg/l	3	2.5
≤3.0 (clear)	64	52.9	**Chromaticity**		
> 3.0 (turbid)	57	47.1	< 15	42	34.7
**Flow or static**			15~30	32	26.4
flow	9	7.4	30~45	32	26.4
static	112	92.6	> 45	15	12.5
**Water depth**			**Emergent plant**		
< 0.5 m	14	11.6	yes	41	33.9
0.5~1.0 m	71	58.7	no	80	66.1
> 1.0 m	36	29.7	**Floating plant**		
**Predator**			yes	94	77.7
yes	67	55.4	no	27	22.3
no	54	44.6			

^1 ^Each potential mosquito breeding sites are classified based on the main characteristics (the types of breeding site, perimeter, distance to the nearest house, substrate types, canopy, debris, the stability of mosquito larval breeding sites, turbidity, flow or static, water depth, predator, algae, pH, COD, ammonia nitrogen, sulphate, chloride, chromaticity, emergent plant, floating plant and so on).

^2 ^N refers to the number of each characteristic.

^3^% refers to the percent composition of the components of each characteristic; the total of percent composition of the components of each characteristic is 100%

Chi-square analysis was used to determine the importance of factors for explaining the presence of *An. sinensis *larvae. There were marked differences in the composition of *An. sinensis *larvae among the types of breeding site (*χ*^2 ^= 18.214, df = 4, P = 0.001), the stability of mosquito larval breeding sites (*χ*^2 ^= 5.356, df = 1, P = 0.021), water depth (*χ*^2 ^= 11.412, df = 2, P = 0.003), chromaticity (*χ*^2 ^= 9.690, df = 3, P = 0.021), predator (*χ*^2 ^= 8.091, df = 1, P = 0.004), pH (*χ*^2 ^= 11.477, df = 3, P = 0.009), COD (*χ*^2 ^= 34.377, df = 3, P < 0.001), ammonia nitrogen (*χ*^2 ^= 57.233, df = 3, P < 0.001), sulphate (*χ*^2 ^= 13.344, df = 3, P = 0.004), and chloride (*χ*^2 ^= 12.461, df = 3, P = 0.006). The primary breeding sites of *An. sinensis *larvae included ponds (43.3%), puddles (38.3%), and river fringes (13.3%). In these breeding sites, most of *An. sinensis *larvae preferred to breed in permanent water bodies (95.0%), with depth (0.5 m ~ 1.0 m) (73.3%), pH (8.0 ~ 10.0) (85%), COD (< 2 mg/L) (75%), ammonia nitrogen (< 0.4 mg/L)(86.7%), sulphate (< 150 mg/L)(58.3%), chloride (15 ~ 30 mg/L)(53.3%), chromaticity (< 45) (88.3%), and no predators (68.3%).

All factors which were of statistical significance using Chi-square analysis were selected and further analyzed by Binary logistic regression analysis. The main parameters entered into the model included the types of breeding site, the stability of mosquito larval breeding site, water depth, predators, pH, COD, ammonia nitrogen, sulphate, chloride, and chromaticity (Table 3). Table 4 demonstrates the key factors that determined the presence of *An. sinensis *larvae in water bodies in the village surroundings by Binary logistic regression analysis. The types of breeding site (OR 0.178; P = 0.006), water depth (OR 0.262; P = 0.037), COD (OR 0.308; P = 0.010), ammonia nitrogen (OR 0.357; P = 0.002), and sulphate (OR 0.413; P = 0.007) were found to be the key factors which determined the presence of *An. sinensis *larvae. For effective *An. sinensis *larvae control in future, these factors should be highly emphasized.

**Table 3:** Positive parameters that determined the presence of *An.sinensis *larvae in water bodies in the village surroundings by Chi-square analysis

Variable	χ^2^	df	**Sig**.
Breeding site type	18.214	4	0.001**
The stability of mosquito larval breeding site	5.356	1	0.021*
Water depth	11.412	2	0.003**
Predator	8.091	1	0.004**
pH	11.477	3	0.009**
COD	34.377	3	0.000**
Ammonia nitrogen	57.233	3	0.000**
Sulphate	13.344	3	0.004**
Chloride	12.461	3	0.006**
Chromaticity	9.690	3	0.021*

Sig-Significant; df-Degrees of freedom;

* P < 0.05, ** P < 0.01

**Table 4:** Parameters that determined the presence of *An.sinensis *larvae in water bodies in the village surroundings by Logistic regression analysis

Variable	B	**S.E**.	Wald	df	**Sig**.	OR	95.0% C.I. for OR	
							
							Lower	Upper
Breeding site type	-1.725	0.631	7.464	1	0.006**	0.178	0.052	0.614
Water depth	-1.338	0.642	4.345	1	0.037*	0.262	0.075	0.923
COD	-1.178	0.458	6.616	1	0.010*	0.308	0.126	0.756
Ammonia nitrogen	-1.029	0.338	9.260	1	0.002**	0.357	0.184	0.693
Sulphate	-0.883	0.326	7.348	1	0.007**	0.413	0.218	0.783
Constant	16.440	3.983	17.039	1	0.000**	1.4E+07		

OR-Odds ratio; C.I.-Confidence interval; Sig-significant; df-Degrees of freedom;

* P < 0.05, ** P < 0.01

## Discussion

The precise identification of the anopheline mosquitoes in Yongcheng city is of great significance to the malaria elimination in China. The rDNA PCR assay revealed that *An. sinensis *was the sole potential anopheles species of Hyrcanus Complex and the finding was identical to the results from previous reports in China [14,47,63]. The river fringes, ponds, puddles, irrigation and drainage ditches in the village surroundings were found to be the main breeding sites of *An. sinensis*, while only 3 containers in the household courtyards, including a plastic bucket only with *An. sinensis *larvae and two other containers (a stone groove and a cement crock). Even though the positive containers were relatively less in sampled containers, these types of containers mentioned above were very popular in the household courtyard of local villages, and thus have epidemiological significance in vector control for malaria elimination in China.

Chi-square analysis demonstrated that factors related to the presence of *An. sinensis *larvae included breeding site type [58], the stability of mosquito larval breeding sites, water depth, predator, pH, COD, ammonia nitrogen, sulphate, chloride and chromaticity respectively. Binary logistic regression analysis revealed that breeding site type, water depth, COD, ammonia nitrogen and sulphate were the key factors that determined the presence of *An. sinensis *larvae. Previous studies found that the breeding site of *An. sinensis *was much the same everywhere [32,64]. It breeds in a wide range of breeding sites which consist of, naturally-made clean water, stagnant or flowing; primary breeding sites include rice fields, ditches, streams, irrigation canals, marshes, ponds, ground pools, among others. In China, it breeds in a wide variety of water collections mainly in paddy fields [26]. Interestingly, there is no paddy field in Yongcheng city. Therefore, ascertaining the precise breeding sites and related water quality index is of significance to the control of *An. sinensis *larvae and the ongoing malaria elimination campaign. Based on the present study, river fringes (13.3%), ponds (43.3%) and puddles (38.3%) were the primary breeding sites of *An. sinensis *larvae. For effective larval control, water bodies such as river fringe, pond and puddle should be given higher priority over others in *An. sinensis *abatement programmes in malaria epidemic areas with *An. sinensis *as the primary vector. This was in conformity with the results presented by similar studies conducted in the areas along the Huang and Huai River in China, with larval breeding sites in small water-bodies, such as ponds, paddy fields or gullies [28,29].

In the current study, 90% of *An. sinensis *larvae were found in permanent breeding sites, such as river fringes, ponds and puddles, while only 10% of *An. sinensis *larvae were found in temporary breeding sites, including roadside ditches, rain pools, shallow drainages, and ground pools. This was coherent with the known preference of most anopheline mosquitoes to breed in natural permanent waters, while only few species were abundant in temporary breeding sites [65]. This could be explained as due to most of the temporary breeding sites being relatively shallow and turbid [59]. Therefore, *An. sinensis *larvae did not adapt to these circumstances. However, it has been reported that large or small temporary ground pools, particularly in the rainy season, could also provide ideal breeding sites for *An. sinensis *[32]. In this study, 73.3% of *An. sinensis *larvae were found in water depths between 0.5 m and 1.0 m, and small amounts of *An. sinensis *larvae (5.0%) were inhabited in water depths less than 0.5 m. Thus, water depth between 0.5 m and 1.0 m might be the ideal breeding depth for *An. sinensis *larvae in Yongcheng city. In the village surroundings, we found the depth of most of the river fringes, ponds, puddles and irrigation drainages and ditches were between 0.5 m and 1.0 m. Therefore, it is also the types of breeding site which should be considered in *An. sinensis *larvae abatement programs in the future.

According to the results of the present study, ammonia nitrogen, COD, and sulphate were negatively correlated to the presence of *An. sinensis *larvae. Ammonia nitrogen is the nutrient of water bodies; however, excess ammonia nitrogen discharged into water bodies could contribute to eutrophication and oxygen depletion in the water, and thus have toxic effects to fish and some aquatic organisms. In the present study, a high level of ammonia nitrogen was observed in the water bodies in the sampled villages, which is likely to lead to oxygen depletion in water bodies, leading eventually to suppressed density of *An. sinensis *larvae. Sulphate is a naturally occurring substance that contains sulphur and oxygen. Sulphate may be leached from the soil and is commonly found in most water supplies. There are several additional sources of sulphate in water. Decaying plant and animal matter may release sulphate into water. The treatment of water with aluminum sulphate (alum) or copper sulphate also introduces sulphate into a water supply. However, excess sulphate has some adverse effects on the water body, fish and some aquatic organisms [66,67]. In the current study, the sulphate level in 30.5% of aquatic sites in the village surroundings was higher than 250 mg/L, and the increased sulphate in water may be explained as the increasing possibility of pollution by domestic sewage, industrial waste water and farmland runoff pollution in Yongcheng City. In environmental chemistry, the COD test is ordinarily used to indirectly measure the amount of organic compounds in water. Most applications of COD determine the amount of organic pollutants found in varieties of surface water or waste water, making COD a useful measure of water quality. In the current study, the COD level in 10.8% aquatic sites in the village surroundings was higher than 3 mg/L, and the increased COD in water may be explained as an increase of organic pollution, and thus may suppress the presence and abundance of *An. sinensis *larvae.

Though other factors, such as the stability of mosquito larval breeding site, predator, pH, chloride, and chromaticity were excluded after being entered into a logistic regression model, their roles could not be ignored. Tadpoles, water bugs, dragonflies and chironomus larvae are suspected to be potential larval predators [68]. When they were found in breeding sites, mosquito larvae were generally absent [59]. Predators may play an important role in regulating populations of *An. gambiae s.l*. in rice paddies, while in permanent larval breeding sites such as ponds, predators such as fishes were observed, and they could suppress the abundance of mosquito larvae [69,70]. In the present study, most of *An. sinensis *larvae were found in water bodies without predators, and this phenomenon could be explained as the suppression effect of predators on *An. sinensis *larvae in the breeding sites.

Based on the findings mentioned above, it is probable that the breeding habit of *An. sinensis *might be changed to some extent. It has previously been demonstrated that mosquito larvae favored a pH-neutral environment [71,72]. However, in the present study, 80.2% of *An. sinensis *larvae lived in water bodies with pH between 8.0 and 10.0 in Yongcheng city, and this was inconsistent with similar studies in China. This phenomenon could be explained as the adaptability of *An. sinensis *larvae to altered environmental conditions, especially water pollution. As to the breeding sites of *An. sinensis*, three containers in the household courtyards discovered the presence of *An. sinensis *larvae. This was the first time that *An. sinensis *larvae were found in containers in the household courtyards in the villages of Yongcheng city. The changes in breeding habit of anopheline mosquitoes were also reported in similar studies. A study in Dar es Salaam, Tanzania, showed a change of *Anopheles sp*. breeding requirements in urban settings during an extended dry period [71,73]. Research in malaria endemic areas in Kuala Lipis, Pahang, Malaysia, demonstrated that changes in breeding characteristics were observed. Instead of breeding in slow flowing streams, most larvae bred in small water pockets along the river margin [73].

Care needs to be taken in interpreting the results of this study. First, this study was conducted during a relatively dry year [74] and the rainfall was relatively low, therefore, it was unclear whether the breeding site structure found during the five months was representative of the structure found in years with normal rainfall. The related parameters of water bodies in normal years might be different from those in the dry years. Second, we did not survey the relatively small breeding sites, such as footprint, wheel mark, and tree hole, because these types of breeding site were uncommon in the field in 2010. For all that, this may lead to the omission of factors potentially important for mosquito control interventions. Third, the present study was done under natural conditions and external factors could play an important role in the colonization and growth of mosquito larvae in these breeding sites. Factors such as total suspended solids (TSS), total dissolved solids (TDS), dissolved oxygen (DO), orthophosphates, biochemical oxygen demand (BOD), heavy metallic elements and their compounds (Mercury, Cadmium, Lead, Arsenic, Chromium, Copper, Zinc, Manganese [75]), radioactive substances, mineral and their compounds contents (Calcium, Potassium, Sodium, Carbonate, Bicarbonate, Ammonium, Nitrate, Phosphate, Magnesium, Sulfate ions), and some microbial contents, were not controlled, which could play a role in the results obtained. Fourth, the urban area was not sampled during the study period, and this may weaken the representativeness of this study to some extent.

## Conclusions

Based on the findings mentioned above, breeding site type, water depth, COD, ammonia nitrogen, and sulphate were found to be the key factors determining the presence of *An. sinensis *larvae in the villages of Yongcheng city. For effective larval control, breeding site type, water depth, COD, ammonia nitrogen, and sulphate should be given higher priority in mosquito abatement programs in malaria endemic areas where *An. sinensis *is the primary vector. These findings could be very useful in understanding the ecology of *An. sinensis *larvae, in planning and implementing of *An. sinensis *larval abatement programs, and in providing strong guarantee for the elimination of malaria in China.

## Abbreviations

COD: Chemical oxygen demand; TSS: Total suspended solids; TDS: Total dissolved solids; DO: Dissolved oxygen; BOD: Biochemical oxygen demand

## Competing interests

The authors declare that they have no competing interests.

## Authors' contributions

The study was conceived by XBL and QYL. XBL, QYL, YHG, and DSR supervised field data collection, analyzed the data and drafted the manuscript. YC assisted with data analysis and together with JLL in writing of manuscript. JYJ, GCZ, HSL, CJZ, HZL and QL assisted with study design and logistical issues. All authors read and approved the final version of manuscript.

## References

[B1] Wu T Nagleand AS Chatterjee AK Road towards new antimalarials- overview of the strategies and their chemical progress Curr Med Chem 2011 18 6 853 871 10.2174/092986711794927748 21182479

[B2] Ramirez JL Garver LS Dimopoulos G Challenges and approaches for mosquito targeted malaria control Curr Mol Med 2009 9 2 116 130 10.2174/156652409787581600 19275622 PMC2925229

[B3] Sachs J Malaney P The economic and social burden of malaria Nature 2002 415 6872 680 685 10.1038/415680a 11832956

[B4] Hay SI Guerra CA Gething PW Patil AP Tatem AJ Noor AM Kabaria CW Manh BH Elyazar IR Brooker S A world malaria map: Plasmodium falciparum endemicity in 2007 PLoS Med 2009 6 3 e1000048 19323591 10.1371/journal.pmed.1000048 PMC2659708

[B5] Kouyate B Sie A Ye M De Allegri M Muller O The great failure of malaria control in Africa: a district perspective from Burkina Faso PLoS Med 2007 4 6 e127. 17550300 10.1371/journal.pmed.0040127 PMC1885453

[B6] Corbel V Henry MC Prevention and control of malaria and sleeping sickness in Africa: where are we and where are we going? Parasit Vectors 2011 4 37 10.1186/1756-3305-4-37 21410946 PMC3065431

[B7] Bousema T Drakeley C Epidemiology and infectivity of Plasmodium falciparum and Plasmodium vivax gametocytes in relation to malaria control and elimination Clin Microbiol Rev 2011 24 2 377 410 10.1128/CMR.00051-10 21482730 PMC3122489

[B8] Mueller I Galinski MR Baird JK Carlton JM Kochar DK Alonso PL del Portillo HA Key gaps in the knowledge of Plasmodium vivax, a neglected human malaria parasite Lancet Infect Dis 2009 9 9 555 566 10.1016/S1473-3099(09)70177-X 19695492

[B9] Guerra CA Snow RW Hay SI Mapping the global extent of malaria in 2005 Trends Parasitol 2006 22 8 353 358 10.1016/j.pt.2006.06.006 16798089 PMC3111076

[B10] Guerra CA Snow RW Hay SI Defining the global spatial limits of malaria transmission in 2005 Adv Parasitol 2006 62 157 179 16647970 10.1016/S0065-308X(05)62005-2 PMC3145102

[B11] Price RN Tjitra E Guerra CA Yeung S White NJ Anstey NM Vivax malaria: neglected and not benign Am J Trop Med Hyg 2007 77 6 Suppl 79 87 18165478 PMC2653940

[B12] Chai JY Re-emerging Plasmodium vivax malaria in the Republic of Korea Korean J Parasitol 1999 37 3 129 143 10.3347/kjp.1999.37.3.129 10507220 PMC2733142

[B13] Tang LH Progress in malaria control in China Chin Med J (Engl) 2000 113 1 89 92 11775219

[B14] Zhou SS Huang F Wang JJ Zhang SS Su YP Tang LH Geographical, meteorological and vectorial factors related to malaria re-emergence in Huang-Huai River of central China Malar J 2010 9 337 10.1186/1475-2875-9-337 21092326 PMC3003275

[B15] Sleigh AC Liu XL Jackson S Li P Shang LY Resurgence of vivax malaria in Henan Province, China Bull World Health Organ 1998 76 3 265 270 9744246 PMC2305715

[B16] Zhou SS Wang Y Fang W Tang LH Malaria situation in the People's Republic of China in 2008 Chin J Parasit Parasitic Dis 2009 27 6 455 457 in Chinese 20232622

[B17] Zhang W Wang L Fang L Ma J Xu Y Jiang J Hui F Wang J Liang S Yang H Spatial analysis of malaria in Anhui province China. Malar J 2008 7 206 10.1186/1475-2875-7-206 PMC2572066 18847489

[B18] Xu BL Su YP Shang LY Zhang HW Malaria control in Henan Province, People's Republic of China Am J Trop Med Hyg 2006 74 4 564 567 16606984

[B19] Takken W Knols BG Malaria vector control: current and future strategies Trends Parasitol 2009 25 3 101 104 10.1016/j.pt.2008.12.002 19168392

[B20] Mendis K Rietveld A Warsame M Bosman A Greenwood B Wernsdorfer WH From malaria control to eradication: The WHO perspective Trop Med Int Health 2009 14 7 802 809 10.1111/j.1365-3156.2009.02287.x 19497083

[B21] Greenwood B Can malaria be eliminated? Trans R Soc Trop Med Hyg 2009 103 Suppl 1 S2 5 19062058 10.1016/j.trstmh.2008.10.027

[B22] Tanner M de Savigny D Malaria eradication back on the table Bull World Health Organ 2008 86 2 82. 10.2471/BLT.07.050633 18297155 PMC2647379

[B23] Baker TD Malaria eradication in India: a failure? Science 2008 319 5870 1616. 18356507 10.1126/science.319.5870.1616d

[B24] Roberts L Enserink M Malaria. Did they really say ... eradication? Science 2007 318 5856 1544 1545 10.1126/science.318.5856.1544 18063766

[B25] Qu FY Historical review on the classification and rectification of Anopheles anthropophagus to An. lesteri in China Chin J Parasit Parasitic Dis 2008 26 3 234 235 in Chinese 19160973

[B26] Chow CY Malaria vectors in China Chin J Entomol 1991 Special Publ 6 67 79 in Chinese

[B27] Sinka ME Bangs MJ Manguin S Chareonviriyaphap T Patil AP Temperley WH Gething PW Elyazar IR Kabaria CW Harbach RE The dominant Anopheles vectors of human malaria in the Asia-Pacific region: occurrence data, distribution maps and bionomic precis Parasit Vectors 2011 4 89 10.1186/1756-3305-4-89 21612587 PMC3127851

[B28] Gou GX Li DF Shang LY Guo XS Wang WX Sui QL Shen YD Hao JL Hu ZT Liang DP The study on ecological habits of Anopheles sinensis in Guantang,Luyi county from 1971 to 1996 Chin J Vector Biol & Control 1998 9 133 134 in Chinese

[B29] Qu CZ Su TZ Vectorial capacity of malaria transmission of Anopheles sinensis in Zhengzhou in nature J Henan Medieal Univ 2000 35 394 396 in Chinese

[B30] Machault V Gadiaga L Vignolles C Jarjaval F Bouzid S Sokhna C Lacaux JP Trape JF Rogier C Pages F Highly focused anopheline breeding sites and malaria transmission in Dakar Malar J 2009 8 138 10.1186/1475-2875-8-138 19552809 PMC2713260

[B31] Gouagna LC Dehecq JS Girod R Boyer S Lemperiere G Fontenille D Spatial and temporal distribution patterns of Anopheles arabiensis breeding sites in La Reunion Island-multi-year trend analysis of historical records from 1996-2009 Parasit Vectors 2011 4 121 10.1186/1756-3305-4-121 21708013 PMC3145585

[B32] Ree HI Studies on Anopheles sinensis, the vector species of vivax malaria in Korea Korean J Parasitol 2005 43 3 75 92 10.3347/kjp.2005.43.3.75 16192749 PMC2712014

[B33] Suarez Sarmiento E Castex Rodriguez M Marquetti MC Martinez C Water pollution and presence of Anopheles albimanus Wiedemann, 1821 and Culex nigripalpus Theobald, 1901 Rev Cubana Med Trop 1990 42 1 130 139 2259776

[B34] Liu XB Liu QY Guo YH Jiang JY Ren DS Zhou GC Zheng CJ Zhang Y Liu JL Li ZF The abundance and host-seeking behavior of culicine species (Diptera: Culicidae) and Anopheles sinensis in Yongcheng city, people's Republic of China Parasit Vectors 2011 4 221 10.1186/1756-3305-4-221 22115320 PMC3267684

[B35] Hunt RH Fuseini G Knowles S Stiles-Ocran J Verster R Kaiser ML Choi KS Koekemoer LL Coetzee M Insecticide resistance in malaria vector mosquitoes at four localities in Ghana, West Africa Parasit Vectors 2011 4 107 10.1186/1756-3305-4-107 21679391 PMC3145582

[B36] Balkew M Ibrahim M Koekemoer LL Brooke BD Engers H Aseffa A Gebre-Michael T Elhassen I Insecticide resistance in Anopheles arabiensis (Diptera: Culicidae) from villages in central, northern and south west Ethiopia and detection of kdr mutation Parasit Vectors 2010 3 1 40. 10.1186/1756-3305-3-40 20416109 PMC2868498

[B37] Goklany IM Climate change and malaria Science 2004 306 5693 55 57 author reply 55-57 10.1126/science.306.5693.55 15459370

[B38] Dye C Reiter P Climate change and malaria: temperatures without fevers? Science 2000 289 5485 1697 1698 11001735

[B39] Thomson MC Doblas-Reyes FJ Mason SJ Hagedorn R Connor SJ Phindela T Morse AP Palmer TN Malaria early warnings based on seasonal climate forecasts from multi-model ensembles Nature 2006 439 7076 576 579 10.1038/nature04503 16452977

[B40] Thomas C Malaria: a changed climate in Africa? Nature 2004 427 6976 690 691 14973466 10.1038/427690b

[B41] Patz JA Hulme M Rosenzweig C Mitchell TD Goldberg RA Githeko AK Lele S McMichael AJ Le Sueur D Climate change: Regional warming and malaria resurgence Nature 2002 420 6916 627 628 discussion 628 10.1038/420627a 12478282

[B42] Hay SI Cox J Rogers DJ Randolph SE Stern DI Shanks GD Myers MF Snow RW Climate change and the resurgence of malaria in the East African highlands Nature 2002 415 6874 905 909 10.1038/415905a 11859368 PMC3164800

[B43] Ototo EN Githeko AK Wanjala CL Scott TW Surveillance of vector populations and malaria transmission during the 2009/10 El Nino event in the western Kenya highlands: opportunities for early detection of malaria hyper-transmission Parasit Vectors 2011 4 144 10.1186/1756-3305-4-144 21781291 PMC3148556

[B44] Gething PW Van Boeckel TP Smith DL Guerra CA Patil AP Snow RW Hay SI Modelling the global constraints of temperature on transmission of Plasmodium falciparum and P. vivax Parasit Vectors 2011 4 92 10.1186/1756-3305-4-92 21615906 PMC3115897

[B45] Imbahale SS Paaijmans KP Mukabana WR van Lammeren R Githeko AK Takken W A longitudinal study on Anopheles mosquito larval abundance in distinct geographical and environmental settings in western Kenya Malar J 2011 10 81 10.1186/1475-2875-10-81 21477340 PMC3080801

[B46] Alemu A Abebe G Tsegaye W Golassa L Climatic variables and malaria transmission dynamics in Jimma town, South West Ethiopia Parasit Vectors 2011 4 30 10.1186/1756-3305-4-30 21366906 PMC3055844

[B47] Zhang HW Su YP Zhou GC Re-emerging malaria in Yongcheng city of Henan province Chin J Vector Biol Control 2007 18 1 42 44 in Chinese

[B48] Service MW Mosquito Ecology-Field Sampling Methods 1993 London: Elsevier Applied Science; Second

[B49] Li L Bian L Yakob L Zhou G Yan G Temporal and spatial stability of Anopheles gambiae larval habitat distribution in Western Kenya highlands Int J Health Geogr 2009 8 70 10.1186/1476-072X-8-70 20021640 PMC2803444

[B50] Gillies MT Coetzee B A supplement to anophelinae of Africa south of Sahara (Afro-tropical region) Pub South Afr Inst of Med Res 1987 55 1 143

[B51] Lu BL Li PS Ji SH Fauna sinica insecta(Diptera: Culicidae) 1997 Beijing, China: Science Press (in Chinese)

[B52] Lee WJ Klein TA Kim HC Choi YM Yoon SH Chang KS Chong ST Lee IY Jones JW Jacobs JS Anopheles kleini, Anopheles pullus, and Anopheles sinensis: potential vectors of Plasmodium vivax in the Republic of Korea J Med Entomol 2007 44 6 1086 1090 10.1603/0022-2585(2007)44[1086:AKAPAA]2.0.CO;2 18047210

[B53] Ma Yajun Qu Fengyi Xu Jiannong Differentiation of Anopheles sinensis and Anopheles anthropophagus using a ribosomal DNA PCR assay Acad J Sec Mil Med Univ 1998 19 3 237 239 in Chinese

[B54] Jeong KY Un S Lee J Lee IY Yong TS Ree HI Population dynamics of five anopheles species of the Hyrcanus group in northern Gyeonggi-do, Korea Korean J Parasitol 2010 48 4 351 353 10.3347/kjp.2010.48.4.351 21234242 PMC3018589

[B55] Oh SS Hur MJ Joo GS Kim ST Go JM Kim YH Lee WG Shin EH Malaria vector surveillance in Ganghwa-do, a malaria-endemic area in the Republic of Korea Korean J Parasitol 2010 48 1 35 41 10.3347/kjp.2010.48.1.35 20333283 PMC2843844

[B56] Minakawa N Mutero CM Githure JI Beier JC Yan G Spatial distribution and habitat characterization of anopheline mosquito larvae in Western Kenya Am J Trop Med Hyg 1999 61 6 1010 1016 10674687 10.4269/ajtmh.1999.61.1010

[B57] Majambere S Fillinger U Sayer DR Green C Lindsay SW Spatial distribution of mosquito larvae and the potential for targeted larval control in The Gambia Am J Trop Med Hyg 2008 79 1 19 27 18606759

[B58] Mwangangi JM Mbogo CM Muturi EJ Nzovu JG Githure JI Yan G Minakawa N Novak R Beier JC Spatial distribution and habitat characterisation of Anopheles larvae along the Kenyan coast J Vector Borne Dis 2007 44 1 44 51 17378216 PMC2731850

[B59] Wanji S Mafo FF Tendongfor N Tanga MC Tchuente E Bilong Bilong CE Njine T Spatial distribution, environmental and physicochemical characterization of Anopheles breeding sites in the Mount Cameroon region J Vector Borne Dis 2009 46 1 75 80 19326712

[B60] Chapman D Water quality assessments. A guide to the use of biota, sediments and water in environmental monitoring 1996 London, UK: Chapman & Hall

[B61] Ho C Chou TC Ch'En TH Hsueh AT The Anopheles hyrcanus group and its relation to malaria in east China Chin Med J 1962 81 71 78 13907849

[B62] Rueda LM Pecor JE Harrison BA Updated distribution records for Anopheles vagus (Diptera: Culicidae) in the Republic of Philippines, and considerations regarding its secondary vector roles in Southeast Asia Trop Biomed 2011 28 1 181 187 21602785

[B63] Zhou GC Zhang HW Su YP Investigation into the results of control of malaria outbreak in Yongcheng County by biological control of mosquito larvae China Trop Med 2009 9 2 228 229+259 in Chinese

[B64] Claborn DM Hshieh PB Roberts DR Klein TA Zeichner BC Andre RG Environmental factors associated with larval habitats of malaria vectors in northern Kyunggi Province, Republic of Korea J Am Mosq Control Assoc 2002 18 3 178 185 12322939

[B65] Awolola TS Oduola AO Obansa JB Chukwurar NJ Unyimadu JP Anopheles gambiae s.s. breeding in polluted water bodies in urban Lagos, southwestern Nigeria J Vector Borne Dis 2007 44 4 241 244 18092529

[B66] Banerjee TK Paul VI Estimation of acute toxicity of ammonium sulphate to the fresh-water catfish, Heteropneustes fossilis. II. A histopathological analysis of the epidermis Biomed Environ Sci 1993 6 1 45 58 8476532

[B67] Butlin KR Adams ME Thomas M Sulphate-reducing bacteria and internal corrosion of ferrous pipes conveying water Nature 1949 163 4131 26. 10.1038/163026a0 18106144

[B68] Kweka EJ Zhou G Gilbreath TM III Afrane Y Nyindo M Githeko AK Yan G Predation efficiency of Anopheles gambiae larvae by aquatic predators in western Kenya highlands Parasit Vectors 2011 4 128 10.1186/1756-3305-4-128 21729269 PMC3141748

[B69] Service MW Mortalities of the immature stages of species B of the Anopheles gambiae complex in Kenya: comparison between rice fields and temporary pools, identification of predators, and effects of insecticidal spraying J Med Entomol 1977 13 4-5 535 545 845895 10.1093/jmedent/13.4-5.535

[B70] Service MW Identification of predators of Anopheles gambiae resting in huts, by the precipitin test Trans R Soc Trop Med Hyg 1973 67 1 33 34 10.1016/0035-9203(73)90305-2 4777420

[B71] Sattler MA Mtasiwa D Kiama M Premji Z Tanner M Killeen GF Lengeler C Habitat characterization and spatial distribution of Anopheles sp. mosquito larvae in Dar es Salaam (Tanzania) during an extended dry period Malar J 2005 4 4 10.1186/1475-2875-4-4 15649333 PMC546229

[B72] Petersen JJ pH as a Factor in Parasitism of Mosquito Larvae by the Mermithid Romanomermis culicivorax J Nematol 1979 11 1 105 106 19305539 PMC2617940

[B73] Rohani A Wan Najdah WM Zamree I Azahari AH Mohd Noor I Rahimi H Lee HL Habitat characterization and mapping of Anopheles maculatus (Theobald) mosquito larvae in malaria endemic areas in Kuala Lipis, Pahang, Malaysia Southeast Asian J Trop Med Pub Health 2010 41 4 821 830 21073056

[B74] Mala AO Irungu LW Shililu JI Muturi EJ Mbogo CC Njagi JK Githure JI Dry season ecology of Anopheles gambiae complex mosquitoes at larval habitats in two traditionally semi-arid villages in Baringo, Kenya Parasit Vectors 2011 4 25 10.1186/1756-3305-4-25 21352608 PMC3060147

[B75] Mireji PO Keating J Hassanali A Mbogo CM Nyambaka H Kahindi S Beier JC Heavy metals in mosquito larval habitats in urban Kisumu and Malindi, Kenya, and their impact Ecotoxicol Environ Saf 2008 70 1 147 153 10.1016/j.ecoenv.2007.03.012 17532467 PMC2673497

